# Preclinical models of stem cell-mediated analgesia and tissue repair: mechanisms, challenges, and future directions

**DOI:** 10.3389/fncel.2026.1787909

**Published:** 2026-03-11

**Authors:** Minshun Zhu, Hao Zhang, Long Liang, Sanbing Wu, Jiaping Chen

**Affiliations:** 1 Department of Rehabilitation, Lu’an Hospital of Traditional Chinese Medicine, Lu’an, China; 2 Department of Orthopedics, Shengzhou Hospital of Traditional Chinese Medicine, Shengzhou, China; 3 Department of Orthopedics, The First Affiliated Hospital of Anhui University of Chinese Medicine, Hefei, China; 4 Department of Orthopedics, Second Affiliated Hospital of Anhui University of Traditional Chinese Medicine, Hefei, China

**Keywords:** analgesia, animal models, damaged tissues, inflammatory responses, intervertebral disc degeneration, nerve regeneration, pain models, preclinical models

## Abstract

As an emerging biological therapeutic approach, stem cell therapy demonstrates broad application prospects in analgesia and tissue regeneration, particularly achieving significant advances in treating conditions such as spinal cord injury and intervertebral disc degeneration. In recent years, preclinical model studies have deepened our understanding of the mechanisms underlying stem cell-mediated pain relief and tissue repair, revealing their potential to regulate inflammatory responses, promote nerve regeneration, and repair damaged tissues through multiple pathways. However, the heterogeneity of preclinical models and the discrepancies between these models and clinical practice, coupled with often insufficient critical appraisal of study quality, remain critical issues requiring urgent resolution in this field. This narrative review systematically summarizes the fundamental theories and key mechanisms underlying stem cell-mediated analgesia and regeneration. It comprehensively evaluates the advantages and limitations of different animal models, critically analyzes major controversies and technical challenges in research, and identifies key directions for future studies. The literature discussed herein was identified through searches in PubMed and Web of Science databases, focusing on recent preclinical studies (primarily within the last decade) involving stem cells, pain models, and tissue regeneration. Selected studies were evaluated for their methodological rigor and contribution to mechanistic understanding. This review aims to synthesize current evidence, critically appraise preclinical models, and provide a forward-looking perspective for research on stem cell-related analgesia and regenerative mechanisms, thereby promoting further development in clinical translation.

## 1 Introduction

Stem cell therapies are gaining increasing prominence in pain management and tissue regeneration, particularly for intractable conditions such as chronic pain and nerve injury. Traditional treatments exhibit significant limitations in alleviating pain and restoring function. For instance, patients with chronic neuropathic pain often develop tolerance and diminished efficacy to opioid medications, accompanied by multiple side effects. This has spurred rapid advancement in stem cell-related research (Meroney et al., 2022; Asgharzade et al., 2020). Stem cells, particularly mesenchymal stem cells (MSCs), have garnered significant attention for their therapeutic potential in these areas (Migliorini et al., 2020; Wang Y. et al., 2025). Among these, adipose-derived stem cells (ADSCs) have demonstrated potential therapeutic value in promoting peripheral nerve regeneration and alleviating neuropathic pain. However, their clinical application remains constrained by regulatory barriers, making the establishment of robust and clinically predictive preclinical models a critical bridge for advancing translational research (Dehdashtian et al., 2020).

As a bridge connecting basic research and clinical application, the design and selection of preclinical models directly impact the reliability of efficacy assessment and the success rate of clinical translation. Existing animal models encompass various pain-related conditions, including peripheral nerve injury, chronic compressive neuropathy, and osteoarthritis (Lee et al., 2022; Yang et al., 2023; Nurul et al., 2021). Additionally, stem cells from diverse sources—such as bone marrow, umbilical cord, and adipose tissue-derived MSCs, as well as induced pluripotent stem cells (iPSCs)—are widely employed in these models to evaluate analgesic and tissue repair effects (Abbaspanah et al., 2021; Owaidah, 2024). These models not only mimic some pathological features of the disease but also enable observation of how stem cells and their secretory products (e.g., exosomes) influence inflammatory responses, nerve regeneration, and immune regulation (Zhang X. et al., 2024; Mussin et al., 2025). However, comparative studies across different animal models reveal that stem cell sources and preparation methods significantly impact therapeutic outcomes, suggesting that preclinical model selection must fully consider disease characteristics and treatment strategies (Zhao and Xie, 2021; Kim et al., 2022). The evolving trends in stem cell therapy highlight its emerging role across various diseases, yet also underscore the need for rigorous preclinical validation (Abusalah et al., 2024).

However, significant variations in models and methodologies employed by different research teams currently exist, leading to inconsistent interpretations of results. Furthermore, some stem cell therapies have encountered inconsistent efficacy and safety issues during clinical translation (Mittal et al., 2025; Shang et al., 2023). For instance, certain clinical studies indicate exacerbated neuropathic pain symptoms following stem cell transplantation, alongside challenges such as immune rejection and low cell survival rates (Shang et al., 2022; Shahrezaei et al., 2025). Furthermore, stem cell preparation, dosage, administration route, and patient variability all influence efficacy, while standardized preparation protocols and quality control remain underdeveloped (Li M. et al., 2022; Waite et al., 2025). These issues highlight the heterogeneity of preclinical models and the inadequacy of evaluation systems (Cao et al., 2021; Jin et al., 2025). While animal models are indispensable tools in medical research, their limitations in fully recapitulating human disease complexity must be acknowledged (Choudhary, 2025). This underscores the urgent need for unified standards and protocols to enhance research comparability and clinical guidance value.

Overall, preclinical model studies on stem cell analgesia and regenerative mechanisms have yielded significant but often preliminary results, providing crucial yet incomplete evidence for the clinical translation of stem cell therapies. Future research should focus on optimizing preclinical model design, promoting multidisciplinary integration, establishing standardized evaluation systems, and deepening investigations into stem cell heterogeneity, immunocompatibility, and long-term safety (Mou et al., 2023). Furthermore, the integration of emerging technologies—such as high-throughput phenotypic screening, gene editing techniques, and Artificial Intelligence (AI)-assisted analysis—will provide robust support for refining and personalizing preclinical models, propelling stem cell analgesia and tissue regeneration toward a new era of precision medicine (Sun et al., 2025; Yang et al., 2025). This review aims to synthesize current knowledge, critically appraise the evidence, and propose a conceptual framework to bridge analgesic and regenerative concepts. We will delineate robust findings from speculative hypotheses, analyze model limitations, and discuss tangible short-term translational pathways.

## 2 Fundamental concepts and mechanistic insights into stem cell analgesia and regeneration

### 2.1 An integrative conceptual framework for analgesia and regeneration

**Figure 1:**
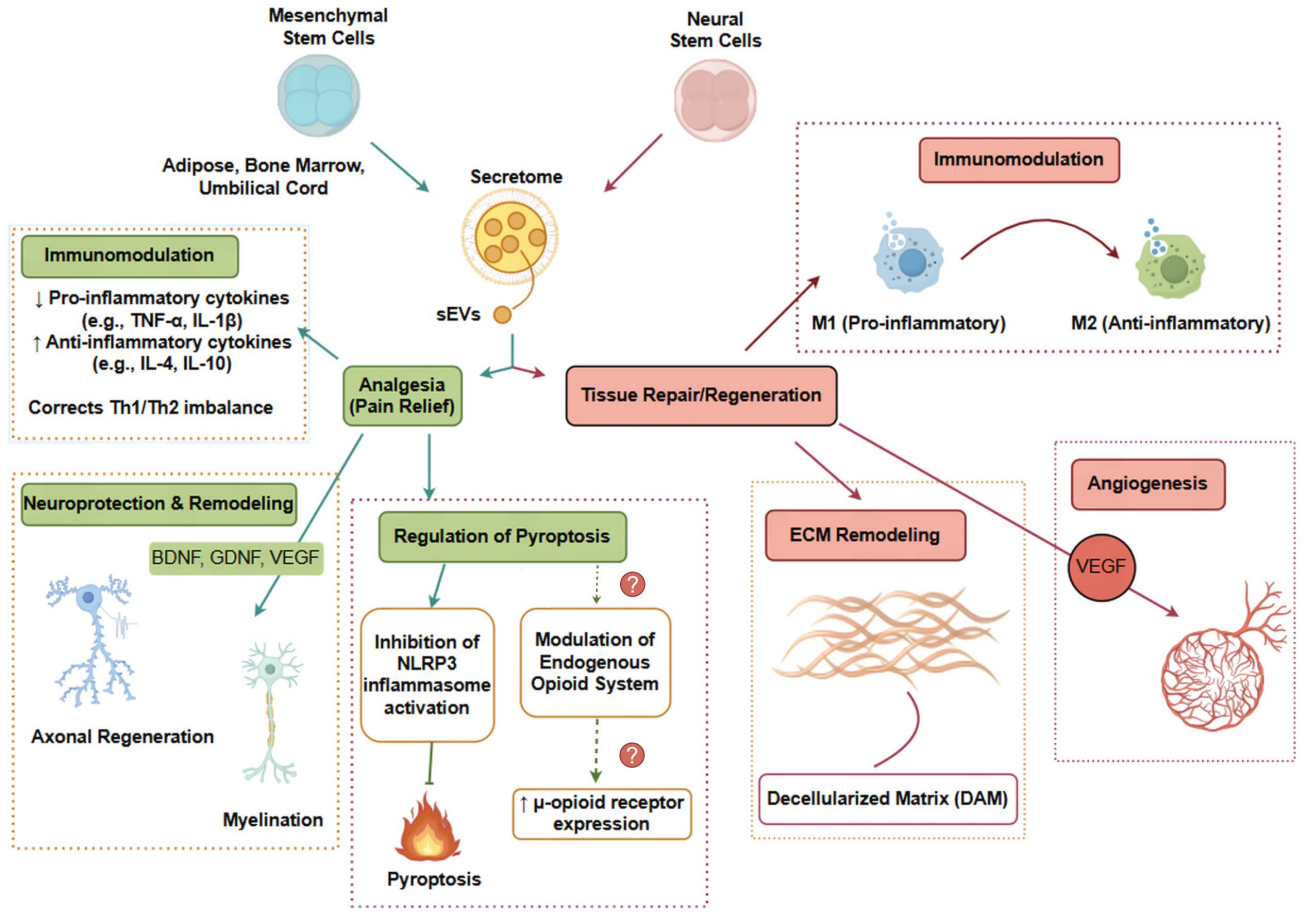
An integrative conceptual framework for stem cell-mediated analgesia and regeneration. This schematic illustrates the proposed unified framework connecting stem cell actions to dual outcomes. Stem cells (MSCs/NSCs) and their extracellular vesicles (EVs) interact with the injury microenvironment through paracrine signaling. Their key mechanisms—immunomodulation, potential regulation of inflammatory cell death (e.g., pyroptosis), trophic support/neural remodeling, and promotion of vascular/ECM remodeling—are interconnected and collectively shift the milieu from a pro-inflammatory, catabolic state to an anti-inflammatory, pro-reparative state. This convergence ultimately leads to pain relief and tissue regeneration. Dashed lines and question marks indicate pathways (e.g., pyroptosis inhibition, opioid modulation) where supportive evidence is primarily correlative, highlighting areas for future causal investigation. sEVs, small extracellular vesicles; BDNF, brain-derived neurotrophic factor; GDNF, glial cell line-derived neurotrophic factor; VEGF, vascular endothelial growth factor; ECM, extracellular matrix. Figure drawn by www.figdraw.com.

The therapeutic effects of stem cells in pain relief and tissue repair are not isolated phenomena but are interconnected through a dynamic interplay with the injury microenvironment. We propose that the core mechanism unifying stem cell-mediated analgesia and regeneration is the multifaceted modulation of the pathological microenvironment toward a pro-repair, anti-inflammatory state. This framework (see Figure 1) posits that stem cells, primarily through paracrine signaling (including via extracellular vesicles, EVs) rather than direct differentiation, act on key cellular targets (immune cells, neurons, glia, endothelial cells, and resident progenitor cells). Their actions converge on four interconnected mechanistic pillars:

1. Immunomodulation: Stem cells exert profound immunomodulatory effects by shifting the balance from pro-inflammatory to anti-inflammatory phenotypes. This involves suppressing the activation and proliferation of pro-inflammatory immune cells (e.g., M1 macrophages, Th1 cells) while promoting regulatory and reparative populations (e.g., M2 macrophages, Treg cells). Concomitantly, they downregulate the release of pro-inflammatory cytokines such as TNF-α, IL-1β, and IL-6, while enhancing anti-inflammatory mediators like IL-4, IL-10, and TGF-β. This immunological rebalancing reduces inflammatory pain drivers and creates a permissive environment for tissue regeneration (Kaka and Modarresi, 2025; Yu and Wang, 2024).
2. Regulation of inflammatory cell death: Emerging evidence indicates that stem cells can mitigate pathological cell death modalities that exacerbate neuroinflammation and pain signaling. In particular, they have been shown to modulate pyroptosis, an inflammatory form of programmed cell death triggered by inflammasome activation (e.g., NLRP3), which leads to cell lysis and massive release of pro-inflammatory cytokines. By suppressing pyroptosis in neurons, glia, and disc cells, stem cells may interrupt the vicious cycle of inflammation and cell death that characterizes chronic pain conditions (Guo et al., 2023).
3. Trophic support and neural plasticity: Stem cells secrete a rich array of neurotrophic factors, including brain-derived neurotrophic factor (BDNF), glial cell line-derived neurotrophic factor (GDNF), nerve growth factor (NGF), and neurotrophin-3 (NT-3). These factors promote neuronal survival, axonal growth, and synaptic plasticity. Importantly, they can inhibit maladaptive plasticity—such as aberrant sprouting of sensory fibers—that contributes to chronic pain, while facilitating appropriate neural network remodeling for functional recovery (Bryk et al., 2022; Zhang et al., 2021).
4. Vascular and extracellular matrix (ECM) remodeling: Effective tissue regeneration requires timely neovascularization to deliver oxygen, nutrients, and circulating reparative cells to the injury site. Stem cells promote angiogenesis through secretion of pro-angiogenic factors such as vascular endothelial growth factor (VEGF), hepatocyte growth factor (HGF), and fibroblast growth factor-2 (FGF-2). They also modulate ECM composition by regulating matrix metalloproteinases (MMPs) and their inhibitors (TIMPs), thereby restructuring the ECM to provide an optimal scaffold for regenerating tissues. Wound healing provides a canonical example highlighting why angiogenesis is indispensable for regeneration; impaired angiogenesis is a hallmark of chronic non-healing wounds, and stem cell-based approaches have been shown to restore pro-angiogenic signaling and accelerate wound closure (Chavez and Gerecht, 2023; Cheng et al., 2025; Yang et al., 2024; Bayraktutan, 2025; Xie et al., 2024; Deng et al., 2025). Importantly, angiogenesis should be evaluated not only by vessel density but also by vessel quality and maturation, as immature or leaky neovessels may fail to support sustained repair. Future studies should incorporate markers of maturation such as pericyte recruitment (e.g., α-SMA/NG2 coverage) and endothelial junction integrity (e.g., VE-cadherin), and consider the balance between ANGPT2-driven sprouting and ANGPT1-mediated stabilization (Yang et al., 2024; Bayraktutan, 2025; Xie et al., 2024).

These four mechanistic pillars are highly interdependent. For example, immunomodulation creates a permissive environment for angiogenesis by reducing inflammatory cytokines that inhibit endothelial cell function. Trophic support and ECM remodeling, in turn, guide the structural integration of newly formed vessels and neural elements. The relative contribution of each pathway likely varies with the disease context, stem cell type, and delivery method. This integrative framework explains how a single therapeutic agent can address both the symptom (pain) and its underlying cause (tissue damage), while also highlighting points of uncertainty—such as the precise molecular triggers of each pathway—that require further validation.

### 2.2 Stem cell types and their analgesic-regenerative potential

Stem cells exhibit immense application potential in analgesia and tissue regeneration due to their unique self-renewal capacity and multipotent differentiation potential. Major stem cell types include neural stem cells (NSCs) and MSCs. Among these, MSCs are the most widely studied in the context of analgesia and tissue regeneration due to their relative ease of isolation, multipotent differentiation potential, and robust paracrine activity (Migliorini et al., 2020; Wang Y. et al., 2025; Erceg Ivkošić et al., 2023). As pluripotent cells within the central nervous system, NSCs can differentiate into neurons, astrocytes, and oligodendrocytes, possessing the ability to repair nerve damage and promote neural regeneration. MSCs derived from diverse sources such as bone marrow, adipose tissue, and synovium possess the potential to differentiate into multiple cell types including chondrocytes, osteoblasts, and adipocytes. Consequently, they are extensively utilized in therapeutic research for conditions like osteoarthritis and nerve injury (Erceg Ivkošić et al., 2023; Li Y. et al., 2022).

As detailed in the integrative framework (Section 2.1), both NSCs and MSCs exert their therapeutic effects primarily through the four core mechanisms of immunomodulation, regulation of inflammatory cell death, trophic support, and vascular-ECM remodeling (Kaka and Modarresi, 2025; Bryk et al., 2022; Almahasneh et al., 2023). For instance, MSCs promote regeneration of damaged neural tissue by secreting multiple cytokines, including stem cell factor, VEGF, and GDNF, which align with the trophic support and vascular remodeling pillars. Simultaneously, they modulate the local immune environment to reduce inflammatory responses, thereby alleviating pain symptoms through the immunomodulatory pillar (Kaka and Modarresi, 2025; Almahasneh et al., 2023). Furthermore, NSCs and MSCs can repair neural pathways and restore neural function by promoting axonal regeneration and myelin formation—processes that depend on both trophic support and a permissive, anti-inflammatory microenvironment (Bryk et al., 2022).

In recent years, extracellular vesicles (EVs), especially small extracellular vesicles (sEVs), have garnered significant attention as key carriers of stem cell functions. sEVs can carry various bioactive molecules such as proteins, nucleic acids, and metabolites, participating in intercellular communication and signaling to regulate pain signal transmission and inflammatory responses (Zhang et al., 2023). MSC-derived sEVs have demonstrated significant analgesic and tissue regenerative effects in multiple pain models, positioning them as a potential alternative or adjunct to stem cell therapy. On a biological level, EVs offer a cell-free therapy that eliminates risks associated with cell viability, uncontrolled differentiation, and vascular occlusion. They may also exhibit lower immunogenicity and possess an inherent ability to cross biological barriers. On a translational level, EVs present advantages in terms of standardization, storage, shelf-life, and potential for engineering to enhance targeting or payload (Wiklander et al., 2019). However, critical challenges remain in large-scale manufacturing, potency standardization, and pharmacokinetic profiling compared to whole cell therapies.

### 2.3 Key pathways and signaling molecules in analgesic mechanisms

Stem cells exert multifaceted molecular actions in analgesia, primarily involving inflammatory factor suppression, neuroprotection, and neural network remodeling. These effects are mediated through specific signaling pathways that operate within the four mechanistic pillars outlined in “Section 2.1.”

Regarding the immunomodulatory pillar, studies indicate that intravenous administration of MSCs effectively alleviates neuropathic pain through anti-inflammatory actions within the central nervous system. For instance, in rat chronic constriction injury models, MSC injection significantly alleviated mechanical and thermal hypersensitivity. Immunohistochemistry revealed that stem cells migrated to the cerebral cortex, expressing markers such as proliferating cell nuclear antigen, CD117, and Nestin, indicating their active participation in neural repair (Kotb et al., 2021). At the molecular level, MSCs suppress pro-inflammatory cytokine release while promoting anti-inflammatory factors like IL-4 and IL-10 by modulating key signaling pathways. Specifically, they have been shown to inhibit the NF-κB pathway in microglia and macrophages, thereby reducing the transcription and release of pro-inflammatory cytokines IL-1β and TNF-*α*, a concept introduced in the immunomodulatory pathway in “Section 2.1” (Yu and Wang, 2024). This indicates that stem cell-mediated analgesia extends beyond the local injury site to involve inflammatory regulation and functional remodeling of neural networks within the central nervous system.

Addressing the regulation of inflammatory cell death pillar, the emerging cell death mechanism pyroptosis has gained attention in pain pathophysiology. Pyroptosis, an inflammatory form of cell death, triggers cell lysis and massive pro-inflammatory cytokine release by activating inflammasomes, particularly the NLRP3 inflammasome and its downstream effector caspase-1, thereby exacerbating neuroinflammation and pain signal transmission. Recent studies reveal elevated expression of pyroptosis-associated molecules like MFN2 in disc degeneration and associated neuropathic pain, accompanied by immune cell infiltration (CD8+ T cells, NK cells, etc.), suggesting pyroptosis may play a crucial role in disease progression and pain development (Guo et al., 2023). Stem cells and their secreted vesicles (e.g., MSC-derived EVs) have been demonstrated to modulate the inflammatory environment by suppressing microglial and astrocytic activation and reducing inflammatory mediator production. This may involve downregulation of the NLRP3/caspase-1/GSDMD signaling axis, thereby potentially alleviating neuroinflammation and pain (Zhang et al., 2021; Zhang et al., 2023). It is crucial to note that the causal link between stem cell therapy, inhibition of pyroptosis, and pain relief *in vivo* is primarily supported by correlative evidence (e.g., co-occurrence of therapy, reduced pyroptosis markers, and behavioral improvement). Dedicated mechanistic studies are needed to establish causality. To establish causality, future studies should incorporate loss-of-function designs. For example, pharmacological blockade of inflammasome/caspase-1 signaling (e.g., caspase-1 inhibition) can be combined with stem cell/EV treatment to test whether pyroptosis suppression is necessary for analgesia. Genetic approaches (e.g., NLRP3- or GSDMD-deficient models) or engineering stem cells/EV cargo to selectively modulate pyroptosis nodes would further strengthen mechanistic inference by demonstrating pathway dependence rather than co-occurrence.

Within the trophic support pillar, stem cells promote neuroprotection and regeneration by secreting growth factors and neurotrophic factors that activate specific intracellular signaling cascades in target cells. For instance, BDNF and GDNF secreted by stem cells bind to their respective receptors (TrkB and GFRα) on neurons, leading to activation of downstream pathways such as ERK/MAPK and PI3K/Akt, which promote neuronal survival, axonal growth, and synaptic plasticity. In a sciatic nerve transection model, neural crest stem cells alleviated hyperalgesia, restored motor function, and suppressed activation of ERK and NF-κB signaling pathways in the spinal cord, thereby reducing central sensitization (Zhang et al., 2021). Furthermore, stem cells may exert analgesic effects in part by modulating the endogenous opioid system, such as potentially enhancing *μ*-opioid receptor expression to amplify endogenous analgesia (Fernandes et al., 2022). This hypothesis requires further direct experimental validation, potentially involving opioid receptor antagonists or receptor knockout models to establish causality.

The vascular remodeling pillar, while critical for tissue regeneration, also indirectly contributes to analgesia by improving tissue perfusion and resolving ischemia-associated pain. Key signaling molecules in this pillar include VEGF, which activates the VEGFR2/PI3K/Akt pathway in endothelial cells to promote angiogenesis, and angiopoietins (ANGPT1/2), which regulate vessel maturation and stabilization via the Tie2 receptor (Chavez and Gerecht, 2023; Cheng et al., 2025). The balance between ANGPT2-driven sprouting and ANGPT1-mediated stabilization is particularly important for generating functional, non-leaky vessels that support long-term tissue repair (Yang et al., 2024; Bayraktutan, 2025; Xie et al., 2024).

In summary, the analgesic and regenerative effects of stem cells are mediated by a network of interconnected signaling pathways that map onto the four mechanistic pillars. NF-κB and NLRP3/caspase-1 are central to immunomodulation and pyroptosis regulation; ERK/MAPK, PI3K/Akt, and TrkB/GFRα signaling underlie trophic support; and VEGF/VEGFR2 and Angpt/Tie2 pathways drive vascular remodeling. Understanding the specific contributions of each pathway in different disease contexts will be essential for optimizing stem cell-based therapies.

## 3 Applications and challenges of preclinical models in stem cell analgesia and regeneration research

### 3.1 A taxonomy of animal models: pain modalities, regeneration endpoints, and translational value

Animal models serve as crucial tools for simulating human disease pathogenesis and therapeutic responses, occupying a central role in preclinical research on stem cell analgesia and regenerative mechanisms. The selection of an appropriate model is paramount and should align with the specific research question, considering the type of pain, the tissue targeted for regeneration, and the desired translational outcome. Table 1 provides a comparative overview of common preclinical models, mapping them to key characteristics. This taxonomy aids in understanding the strengths and limitations of each model system.

**Table 1:** Taxonomy of preclinical models for stem cell analgesia and regeneration research.*

Disease/injury	Example models	Primary pain modality simulated	Key regeneration endpoints	Strengths / translational value	Major limitations / translational gaps
Peripheral Neuropathy	Chronic Constriction Injury (CCI); Spared Nerve Injury (SNI)	Mechanical/thermal allodynia, hyperalgesia	Nerve fiber regeneration, myelination, functional sensorimotor recovery	Well-established, robust behavioral readouts, good for screening analgesic efficacy.	Poorly models spontaneous pain; species differences in nerve anatomy/regeneration.
Spinal Cord Injury (SCI)	Contusion, Compression, Transection	Central neuropathic pain (allodynia, hyperalgesia), spontaneous pain (limited)	Axonal sprouting, remyelination, glial scar modulation, locomotor recovery.	Models key aspects of trauma and secondary damage; locomotor scales (BBB) are standardized.	Difficulty modeling chronic pain complexity; rodent vs. human neuroanatomy differences; locomotor recovery may not equate to pain relief.
Intervertebral Disc Degeneration (IDD)	Needle puncture; Chemically-induced (e.g., chondroitinase)	Radicular/compressive pain (indirectly via evoked hypersensitivity)	Disc height, matrix (aggrecan, collagen II) restoration, nucleus pulposus cell viability.	Allows study of structural repair in a complex tissue.	Evoked pain may not mimic chronic low back pain; large animal models needed for biomechanical relevance.
Osteoarthritis (OA)	Medial Meniscal Destabilization (DMM); Mono-iodoacetate (MIA) injection	Movement-evoked and weight-bearing pain, joint hypersensitivity.	Cartilage integrity, subchondral bone remodeling, synovitis reduction.	Models progressive joint degeneration and pain-related disability.	Species differences in joint loading and cartilage repair capacity.

*This classification summarizes the general attributes, applications, and limitations of common preclinical models as discussed in the relevant literature (Lee et al., 2022; Yang et al., 2023; Nurul et al., 2021; Choudhary, 2025; Yu and Wang, 2024; Cheng et al., 2021; Teixeira-Santos et al., 2023; Malli et al., 2021).

This table provides a taxonomic overview of prevalent preclinical animal models, delineating their corresponding pain modalities, key regeneration endpoints, and translational value to inform rational model selection in stem cell-based analgesia and regeneration research.

To provide necessary context, it is important to recognize that preclinical pain research employs a wide spectrum of animal models designed to mimic the diverse etiologies of human pain conditions. These can be broadly categorized into inflammatory pain models [e.g., Complete Freund‘s Adjuvant (CFA)-induced arthritis), neuropathic pain models (e.g., chronic constriction injury (CCI), spinal nerve ligation (SNL), spared nerve injury (SNI)], incisional pain models (post-operative pain), and cancer-induced bone pain models (Cheng et al., 2021; Teixeira-Santos et al., 2023). The choice of model is dictated by the specific clinical condition being targeted.

In this review, we have deliberately focused on models of spinal cord injury (SCI) and intervertebral disc degeneration (IDD) for several reasons. First, these conditions represent a significant clinical burden where the dual therapeutic potential of stem cells—both analgesia and tissue regeneration—is most critically needed. Second, research in SCI and IDD has yielded a relatively mature body of preclinical evidence, allowing for a more substantive discussion of mechanisms and challenges. Third, they serve as paradigmatic examples of neuro-orthopedic disorders where pain is intricately linked to structural damage, thus providing an ideal framework to explore the interconnected mechanisms of analgesia and regeneration proposed in Section 2. While other valuable models exist, focusing on SCI and IDD enables a deeper, more coherent analysis within the scope of this narrative review.

For common neuro-orthopedic conditions such as IDD, SCI, models strive to replicate pathological features and pain manifestations. IDD animal models are predominantly established through mechanical injury, chemical induction, or genetic engineering methods to induce structural disruption and cellular changes in the intervertebral disc, thereby mimicking the primary etiology of human low back pain. Physical injury techniques such as puncture or excision can induce disc degeneration in various animals including mice, rats, and even rabbits. Histologically, these models exhibit annular tears, nucleus pulposus extrusion, and apoptosis, accompanied by inflammatory cell infiltration and abnormal cytokine expression. However, due to anatomical differences between animal and human discs—such as variations in cellular composition and load-bearing mechanisms—the pathological changes and pain behaviors observed in these models have limitations when compared to humans (Malli et al., 2021; Peng et al., 2021). Regarding pain behavior assessment, commonly used mechanical and thermal sensitivity tests reflect animal hyperalgesia states but often fail to fully correspond to the complex experience of human chronic low back pain, particularly spontaneous pain and affective components.

SCI models typically involve physical compression, contusion, or transection of the spinal cord to mimic clinical spinal trauma. These models induce pathological processes in animals following acute SCI, including loss of neurological function, inflammatory responses, and failure of neural regeneration. Motor dysfunction and sensory abnormalities in animals can be assessed using behavioral scoring systems like the Basso, Beattie, Bresnahan (BBB) scale and thermal stimulation tests, which reasonably reflect functional deficits and some pain states following human SCI (Mukherjee et al., 2022). However, these models remain inadequate in reproducing the full complexity of chronic neuropathic pain following human SCI, particularly its multidimensional manifestations such as spontaneous pain and allodynia. Blast SCI models, while relevant for specific trauma types, add further heterogeneity (Yu and Wang, 2024).

### 3.2 Model heterogeneity and its impact on research outcomes: a critical appraisal

In preclinical model studies of stem cell analgesia and regenerative mechanisms, model heterogeneity significantly affects the stability and reproducibility of research findings. Differences in experimental design, animal species, and injury induction methods often lead to substantial variability in evaluating stem cell efficacy. First, distinct physiological structures and immune responses across animal species—such as mice, rats, or larger mammals—directly impact stem cell survival, migration, and the expression of analgesic and regenerative functions. For instance, the distribution of *μ*-opioid receptors (MOR), critical for analgesia and respiratory regulation in the mammalian brainstem, exhibits species specificity, potentially affecting stem cells’ ability to modulate pain and respiratory function (Lynch et al., 2023). While Sprague–Dawley (SD) rats and guinea pigs (as mentioned in Table 1) are commonly used due to their size, cost-effectiveness, and well-characterized behavior, the field utilizes a broader range of models. Mice, particularly transgenic lines, are indispensable for mechanistic studies involving specific genes or immune cell lineages (e.g., using NLRP3 knockout mice to study pyroptosis) (Pulendran et al., 2010). Rabbits are frequently employed in orthopedic research, especially for IDD and cartilage defects, owing to their larger disc and joint sizes, which facilitate surgical manipulation and imaging (Zhou et al., 2024). For pre-clinical validation closer to human application, large animal models such as pigs, goats, and non-human primates are increasingly utilized. Their anatomy, joint loading, and immune systems more closely resemble humans, providing critical data on safety, dosing, and biomechanical integration before clinical trials (Jiang et al., 2023; Liu et al., 2022; McClements et al., 2020; Li et al., 2021). This phylogenetic diversity, from rodents to large mammals, while essential for translational de-risking, is a primary source of model heterogeneity. Differences in disc cellularity, spinal cord anatomy, and immune responses across these species can lead to divergent outcomes when testing the same stem cell product, thereby contributing to the difficulty in translational research (Lynch et al., 2023; Chan et al., 2023). Secondly, injury induction methods—such as neurotrauma, inflammatory models, or chemically induced models—differ in their pathological mechanisms and inflammatory environments, leading to distinct mechanisms of stem cell action across models and further increasing result heterogeneity. Furthermore, factors in experimental design—such as stem cell administration routes, dosages, and time windows—can all contribute to therapeutic efficacy variations. Crucially, many preclinical studies suffer from small sample sizes, lack of blinding, and inadequate statistical power, which threaten internal validity and can lead to overestimation of effect sizes (Landis et al., 2012). Significant controversy exists in the literature regarding model selection: some studies advocate for complex models closer to human pathologies to enhance clinical translational value, while others favor simplified models with high standardization and reproducibility to ensure comparability of results and clarity in mechanism elucidation. This controversy directly impacts the evaluation of stem cell efficacy and the depth of mechanism research. In summary, model heterogeneity is not only a variable that cannot be ignored in studies of stem cell analgesia and regeneration mechanisms, but also requires researchers to fully consider the rationality of model selection and the standardization of experimental conditions when designing experiments, in order to more accurately assess the potential and mechanisms of stem cell therapy (Lynch et al., 2023; Chan et al., 2023; Chiang et al., 2016; Miyano et al., 2022; Zhang et al., 2014; He et al., 2020). As illustrated in Table 2, substantial heterogeneity exists across preclinical stem cell analgesia studies with respect to species, injury induction paradigms, stem cell source (human vs. rodent), delivery route (intravenous vs. intrathecal vs. intra-articular), dosing regimens, and selected outcome measures. These methodological differences significantly complicate cross-study comparisons and may contribute to divergent conclusions regarding therapeutic efficacy.

**Table 2:** Heterogeneity in preclinical study design: a comparison of key parameters across selected stem cell analgesia studies.

Pain / injury model	Animal species / strain	Stem cell type and source	Administration route and dose	Key efficacy endpoints	Reference
Chronic Constriction Injury (CCI) of the sciatic nerve (four loose ligatures with 3–0 chromic gut sutures)	Sprague–Dawley rats	Human amniotic fluid–derived mesenchymal stem cells (hAFMSCs)	Intravenous (IV) injection; 5 × 10^5^ cells, administered once daily for 3 consecutive days	Mechanical withdrawal threshold (von Frey); thermal withdrawal latency; CatWalk XT gait analysis; inflammatory and neural markers in sciatic nerve and DRG	Pulendran et al. (2010)
Partial Sciatic Nerve Ligation (PSNL)	Sprague–Dawley rat (5 weeks)	Human adipose-derived MSCs (AD-MSCs) and human umbilical cord-derived MSCs (UC-MSCs)	Intravenous (tail vein) 1 × 10^6^ cells per rat, administered 4 days post-PSNL	Mechanical hypersensitivity (von Frey), weight-bearing asymmetry; Iba-1 & ATF-3 expression; myelin basic protein recovery	Zhou et al. (2024)
Spinal Nerve Ligation (SNL; L5 ligation)	Sprague–Dawley rats	Rat mesenchymal stem cells (rMSCs)	Intrathecal injection; 1 × 10^5^ cells in 10 μL, administered on day 7 after ligation	Mechanical allodynia (von Frey); reactive oxygen species (DHE staining); spinal inflammatory markers	Jiang et al. (2023)
Monosodium Iodoacetate (MIA)-induced Osteoarthritis (OA)	10-week-old male Sprague–Dawley rats	Bone marrow MSC-derived exosomes (BMSC-EXOs)	Intra-articular injection; 40 μg in 100 μL, once weekly for 6 weeks	Paw withdrawal threshold (PWT); paw withdrawal latency (PWL); DRG expression of CGRP and iNOS; histological scoring (OARSI)	Liu et al. (2022)

This table systematically contrasts core experimental variables across representative analgesia studies to demonstrate how heterogeneity in animal species, injury models, stem cell sources, delivery routes, dosing schedules, and selected endpoints may influence reported therapeutic efficacy. The marked variability highlighted here reinforces the challenges in cross-study comparability and meta-analytic synthesis, and supports the necessity for standardized methodological frameworks in stem cell analgesia research.

### 3.3 Diversity of evaluation indicators and technical approaches: limitations and overestimation of efficacy

Preclinical model studies on stem cell analgesia and regenerative mechanisms involve multidimensional evaluation indicators and technical approaches, encompassing behavioral testing, biochemical markers, immunohistochemistry, and imaging techniques, forming a relatively comprehensive evaluation system. Behavioral testing serves as a crucial method for evaluating stem cell therapy in neuropathic pain and tissue injury functional recovery. Common approaches include mechanical pain threshold testing (e.g., von Frey filaments, electronic von Frey), gait analysis, and dynamic weight-bearing tests (Lee et al., 2023). These methods provide direct insights into evoked pain perception and motor function changes in animal models. However, a major limitation is their inability to adequately capture key dimensions of the human pain experience, particularly spontaneous (ongoing) pain and the affective/emotional component of suffering. This gap can lead to a significant overestimation of analgesic efficacy if a therapy reduces hypersensitivity but does not alleviate spontaneous pain. Most standard tests measure hypersensitivity to external stimuli (allodynia/hyperalgesia). Specialized tests (e.g., conditioned place preference/aversion, facial grimace scales, burrowing behavior) are being developed to address this, but are not yet standard (Negus, 2019).

#### 3.3.1 Beyond evoked hypersensitivity: assessing spontaneous and affective pain

A major limitation of traditional evoked assays (e.g., von Frey and Hargreaves) is their incomplete representation of the human pain experience, particularly spontaneous (ongoing) pain and the affective/motivational dimension of suffering (Ronis et al., 2020). This mismatch can lead to an overestimation of therapeutic benefit when hypersensitivity is reduced without meaningful relief of ongoing pain. Accordingly, incorporation of complementary endpoints is increasingly recommended. Facial grimace scales provide a non-invasive readout of spontaneous pain but require rater training and may be confounded by sickness behavior (Mota-Rojas et al., 2025). Conditioned place preference/avoidance (CPP/CPA) more directly captures the affective component of pain relief, yet depends on learning and memory and can be confounded in CNS injury models (Tappe-Theodor et al., 2019). Burrowing and other ethologically relevant behaviors are sensitive to inflammatory and ongoing pain and are simple to quantify, but can be influenced by strain differences and motor deficits (Bryden et al., 2015). Therefore, future preclinical studies should adopt a battery approach combining evoked hypersensitivity with at least one spontaneous/affective measure (e.g., von Frey + grimace + burrowing), thereby improving translational validity and reducing efficacy overestimation.

Regarding biochemical indicators, inflammatory cytokines (e.g., TNF-α, IL-1β, IL-6), neurotransmitter-related proteins (CGRP, TRPA1, etc.), and pain-associated molecules (P2X4, P2X7) are extensively monitored to elucidate stem cell therapy’s regulatory effects on the inflammatory environment and analgesic mechanisms (Kan et al., 2022; Moore et al., 2022). Immunohistochemistry is employed to visualize stem cell colonization, differentiation, and tissue repair processes. Specific markers (e.g., SOX9, TGF-β, Col2a1) provide direct visualization of tissue regeneration and cellular phenotype changes, commonly used to validate tissue regeneration in cartilage, intervertebral discs, and other tissues (Khalid et al., 2023; Ekram et al., 2021). Additionally, advanced imaging techniques like magnetic resonance imaging (MRI) and micro-computed tomography (micro-CT) provide crucial non-invasive tools for dynamically monitoring stem cell effects, enabling assessment of tissue structural recovery alongside stem cell survival and distribution (Shelat et al., 2020; Shu et al., 2025).

**Figure 2:**
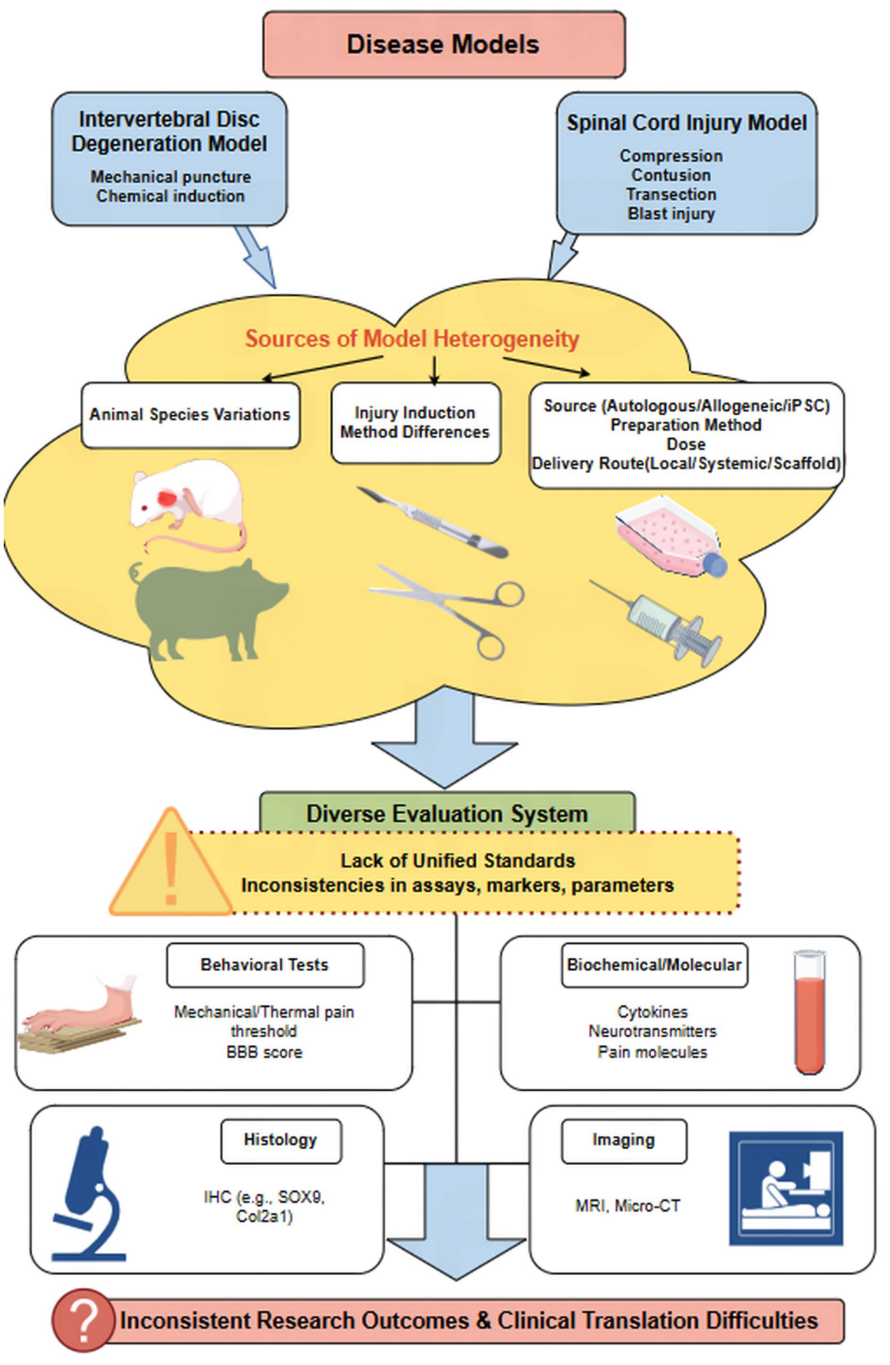
Sources of heterogeneity and standardization needs in preclinical stem cell research. This flowchart maps the pipeline from disease modeling to clinical translation, emphasizing points of variability that contribute to inconsistent outcomes. Key sources of heterogeneity include animal species/strain selection, injury model induction method, and stem cell product variables (source, preparation, dose, delivery route). The evaluation phase employs diverse but often non-standardized methods across behavioral, molecular, histological, and imaging domains. The cumulative effect of this heterogeneity, in the absence of unified standards (e.g., for pain assessment, cell potency), creates a “reproducibility gap” that hinders reliable interpretation and confident clinical translation. Figure drawn by www.figdraw.com.

However, this field currently faces the challenge of lacking standardized evaluation systems. Significant constraints on the comparability of research outcomes arise from variations in animal models, stem cell types, administration methods, and assessment metrics across different studies. For instance, in IDD models, differences exist in the behavioral pain testing methods and scoring criteria used by different laboratories, coupled with inconsistent selection of inflammatory and regenerative molecular markers, making effective cross-laboratory comparisons difficult (Peng et al., 2021; Alini et al., 2023). Similarly, in cartilage regeneration research, while MRI T2 mapping and histological staining are commonly employed, the lack of standardized quantitative analysis parameters and time point settings compromises the accuracy and reproducibility of efficacy assessments (Ford et al., 2020; Freitag et al., 2020). Immunohistochemical techniques also exhibit variations in antibody selection, staining conditions, and result interpretation, further contributing to data inconsistency. Additionally, behavioral testing is highly susceptible to experimental environment and operator influence, with a lack of objective automated analysis tools increasing subjective error (Alini et al., 2023). The integration of AI for automated analysis of behavioral videos, histological images, and complex omics data holds promise for increasing objectivity and uncovering subtle patterns, as explored in related biomedical fields (Choudhary et al., 2025; Priyanka et al., 2023a). The considerable variability in behavioral testing protocols, biomarker selection, and imaging parameters across different laboratories severely limits data integration and cross-study comparisons. The cumulative impact of the model heterogeneity and the lack of standardized evaluation protocols—key themes discussed throughout Section 3—is schematically represented in Figure 2. This flowchart traces the entire preclinical pipeline, from disease modeling and stem cell preparation to delivery and multimodal assessment. It pinpoints the major sources of methodological variability at each stage that collectively contribute to the ‘reproducibility gap’ and hinder reliable clinical translation. Addressing these variabilities through harmonized protocols is a prerequisite for advancing the field.

## 4 Core challenges and controversies in preclinical stem cell therapy research

### 4.1 Differences in stem cell sources and preparation methods: implications for safety

Stem cells originate from diverse sources, primarily including autologous stem cells, allogeneic stem cells, and iPSCs. Significant differences and controversies exist regarding the safety, immunogenicity, and efficacy of stem cells from different sources. A refined threat level analysis is warranted. MSCs derived from adult tissues (e.g., bone marrow, adipose) are generally considered to have low tumorigenic risk, but may pose risks of immunogenicity (especially allogeneic), ectopic tissue formation, or pro-fibrotic effects depending on the microenvironment. NSCs carry a theoretical risk of uncontrolled neural overgrowth or tumor formation (e.g., teratoma from residual pluripotent cells), though advanced purification mitigates this. iPSCs present the highest tumorigenic potential due to the use of reprogramming factors and the need for precise differentiation; however, they offer an unlimited, patient-specific source. EVs, as acellular derivatives, virtually eliminate risks of tumorigenicity and rejection associated with whole cells, but their long-term biodistribution, toxicity profiles, and batch-to-batch variability require further study (Zhang et al., 2023; Li et al., 2024; Zhao et al., 2022; Wang et al., 2022; Bharuka and Reche, 2024).

The diversity of preparation methods and lack of standardization also significantly impact the reproducibility of stem cell research outcomes. Stem cell preparation involves multiple stages—collection, isolation, culture expansion, purification, and activity testing—where variations in any step may compromise the quality and function of the final cell product. For example, the isolation and culture conditions of ADSCs, such as the use of blood products (platelet lysate, plasma, etc.) as culture supplements, significantly affect cell proliferation and differentiation potential. Moreover, the preparation methods and concentrations of different blood products exert varying effects on cell activity (Neubauer et al., 2022; Neubauer et al., 2019). Furthermore, the types and concentrations of growth factors in cell culture media, the culture system (two-dimensional or three-dimensional culture), and the number of cell passages all influence the phenotype and function of stem cells (Sherley, 2023; Kim et al., 2020). Currently, clinical and basic research lack unified standards for cell purity and activity testing, making it difficult to directly compare data across different studies and limiting the clinical translation of stem cell therapies (Lee et al., 2021; Rodríguez-Fuentes et al., 2021).

### 4.2 Controversy over stem cell transplantation methods and dose optimization

Stem cell transplantation administration routes primarily include local injection (e.g., intra-tissue or subcapsular injection), systemic infusion (intravenous or arterial administration), and scaffold-based delivery. Significant differences exist among these routes regarding cell survival rates, targeting specificity, therapeutic efficacy, and safety profiles. For short-term clinical translation, the most believable indications and routes likely involve localized, minimally invasive delivery for focal disorders. For example, intradiscal injection of MSCs/EVs for early-stage discogenic pain, or epidural/intrathecal delivery for focal neuropathic pain, represent plausible near-future pathways. Intra-articular injection for osteoarthritis is another direct route. Systemic infusion for widespread pain (e.g., complex regional pain syndrome) is more speculative due to targeting and safety hurdles (Kabat et al., 2020).

### 4.3 Safety and monitoring considerations are essential when comparing delivery routes

Intravenous infusion is subject to a pulmonary first-pass effect in which a substantial proportion of administered cells may be transiently retained within the lung microvasculature, raising concerns regarding embolic or inflammatory reactions (Cottle et al., 2022). In clinical translation, this necessitates monitoring for infusion reactions and respiratory compromise (e.g., oxygen saturation, respiratory rate, and hemodynamic status). Local delivery routes (e.g., intrathecal/epidural, intra-articular, intradiscal) reduce systemic exposure but introduce procedure- and site-specific risks, including transient neuroinflammation, aseptic meningitis-like reactions, post-dural puncture headache (neuraxial routes), or local synovitis/infection (joint/disc routes). Therefore, standardized adverse-event reporting and predefined route-specific monitoring parameters should accompany efficacy assessment.

**Figure 3:**
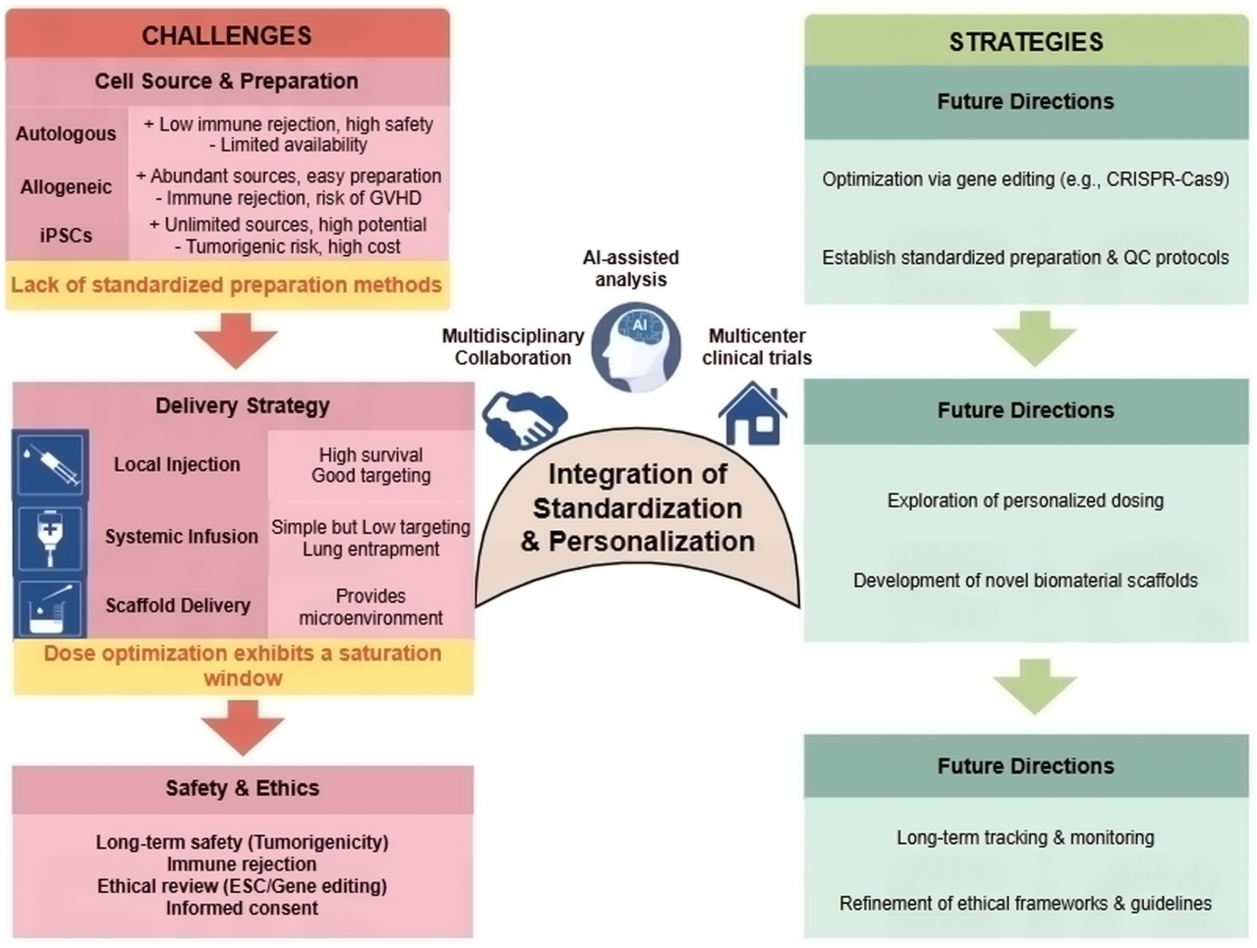
Comparative analysis of challenges and strategic optimizations for stem cell clinical translation. This figure contrasts major translational roadblocks with proposed solution-focused strategies. Challenges are categorized into: (1) Cell source & preparation (heterogeneity, tumorigenicity/immunogenicity risks varying by cell type, lack of standards); (2) Delivery & dosage (route-dependent efficacy, narrow therapeutic window/saturation effect); and (3) Safety & ethics (long-term monitoring gaps, ethical complexities). Corresponding optimization strategies propose: (1) Genetic engineering, standardized manufacturing/QC; (2) Biomaterial-assisted delivery, personalized dosing regimens; and (3) Enhanced long-term surveillance and robust ethical frameworks. The central bridge emphasizes that integrating standardization with personalization, enabled by multidisciplinary collaboration, AI, and multicenter trials, is key to overcoming these hurdles. Figure drawn by www.figdraw.com.

Optimizing stem cell transplantation dosage is a critical factor influencing therapeutic outcomes, with dose-dependent effects exhibiting complex characteristics across different disease models and clinical applications. The concept of a “dose saturation window” is evident, where benefits plateau or even decline beyond an optimal range (Chen et al., 2025; Won et al., 2021). Although formal dose–response curves remain relatively rare in the stem cell/EV analgesia literature, the available dose-ranging studies are broadly consistent with a plateau phenomenon, supporting the concept of a “dose saturation window” rather than a monotonic more-is-better relationship (Robinson et al., 2017; Lee et al., 2018). Optimizing transplantation dose is a critical determinant of therapeutic outcome, and evidence across both preclinical and clinical contexts supports a “dose saturation window,” in which benefits plateau—or may even decline—beyond an effective range (Table 3). These observations argue for formal dose-finding designs rather than assuming a monotonic “more-is-better” relationship. In hematopoietic stem cell transplantation, the impact of CD34+ cell dose is particularly well-defined. Multiple clinical studies indicate that CD34+ cell doses exceeding a certain threshold (e.g., 2.5 × 10^6^/kg) correlate with improved lymphocyte recovery, lower non-relapse mortality, and better overall survival (Gauntner et al., 2022; Pasvolsky et al., 2024; Al-Share et al., 2022). However, excessively high CD34 + cell doses have also been found in some studies to potentially increase the risk of chronic GVHD and even reduce survival rates (Chung et al., 2025), underscoring the need to balance healing promotion with immune-related side effects when optimizing doses. Recent studies on ATG dosing in allogeneic transplantation also indicate that intermediate-dose ATG (7.5 mg/kg) better balances acute GVHD prevention and infection risk compared to lower (6 mg/kg) or higher (9 mg/kg) doses (Zhou et al., 2020). The major translational roadblocks detailed in this section—encompassing cell source heterogeneity, delivery and dosing complexities, and safety/ethical concerns—are juxtaposed with corresponding solution-oriented strategies in Figure 3. This comparative framework highlights how the integration of standardization and personalization, supported by multidisciplinary collaboration and technological innovation, offers a roadmap for navigating the path from preclinical challenges to successful clinical application, as will be elaborated in the following section.

**Table 3:** Representative evidence supporting a “dose saturation window” in stem cell/EV-based analgesia and regeneration.

Cell / product type	Disease / model	Doses tested	Observed dose–response / saturation evidence	Implications for therapy	References
Human BM-MSCs	TNBS-induced colitis (guinea pig) (inflammation + enteric neuropathy repair)	1 × 10^5^ vs. 1 × 10^6^ vs. 3 × 10^6^ cells (local delivery; enema)	1 × 10^5^ showed limited benefit and did not prevent myenteric neuronal loss; 1 × 10^6^ significantly attenuated neuronal loss and inflammation; 3 × 10^6^ produced similar efficacy to 1 × 10^6^ with no further improvement, consistent with a plateau/saturation above ~1 × 10^6^.	Demonstrates a minimum effective dose and plateau: increasing dose beyond an optimal range may not increase engraftment/benefit; supports designing dose-finding (not “more is better”).	Sherley (2023)
Adult human multipotent neural cells (ahMNCs)	Spinal cord injury (rodent SCI)	3 × 10^5^ (low), 1 × 10^6^ (mid), 3 × 10^6^ (high)	Mid-dose 1 × 10^6^ produced significant functional improvement vs. low dose; 3 × 10^6^ did not yield clear additional benefit over 1 × 10^6^, consistent with saturation at higher dose.	Supports an optimal therapeutic window in CNS cell therapy, where escalating dose may not proportionally increase repair due to microenvironment limits (cell survival, niche capacity, diffusion, immune clearance).	Kim et al. (2020)
CD34+hematopoietic stem cells (PBSC graft; clinical allo-HCT)	Allogeneic HCT (adults; PBSC)	Stratified by graft CD34+ cell dose (e.g., higher vs. lower, including >7.5 × 10^6^/kg in outcome analyses)	Higher CD34+ dose associated with better OS/engraftment in some strata, but increased chronic GVHD risk observed with higher dosing—suggesting benefit–risk tradeoff and a narrower “best range” rather than monotonic improvement.	Illustrates a classic dose window in transplantation: cell dose can improve recovery but may increase immune complications; strengthens your “saturation window” argument with clinical-grade evidence.	Lee et al. (2021)
MSCs (clinical-trial landscape; IV delivery, multiple indications)	Across 914 MSC trials (ClinicalTrials.gov analysis; human trials)	Evaluated reported IV dosing distributions; efficacy reported in subsets	Dose–response signals in positive IV trials suggested a relatively narrow effective range; both lower and higher doses could be less effective in limited datasets, consistent with a practical MED/plateau concept at population level.	Supports your manuscript’s translational message: early-phase studies should identify minimal effective dose and avoid untested escalation.	Wang et al. (2022)

This table consolidates empirical evidence from preclinical studies, demonstrating the dose-dependent efficacy and the prevalent saturation effect of stem cell therapies across diverse disease models, which is critical for optimizing therapeutic protocols.

## 5 Current research gaps and potential future directions

### 5.1 Development of more precise and clinically relevant animal models

Future models should better integrate multifactorial pathology. AI-assisted modeling and analysis of anatomical and physiological data from animals can help design more human-relevant injury paradigms and interpret complex outcomes (Choudhary et al., 2025). Beyond multifactorial pathology integration, future models should prioritize the recapitulation of spontaneous and affective pain dimensions, a major limitation of current assays (see “Section 3.3.1”). The development of operant behavioral tasks (e.g., voluntary wheel running, place escape/avoidance paradigms) in the context of these refined injury models will provide a more holistic assessment of stem cell efficacy (Negus et al., 2021). Furthermore, the incorporation of humanized immune system (HIS) mice xenografted with human stem cells can offer invaluable insights into human-specific immune responses and alloreactivity, bridging a critical gap between rodent studies and clinical reality (Zhang T. et al., 2024). Humanized models and large animal studies are crucial for assessing immunocompatibility and functional scale-up (Jiang et al., 2023; Liu et al., 2022).

### 5.2 In-depth analysis of stem cell functional mechanisms

Moving beyond descriptive observations to establish causal mechanistic links is paramount. While single-cell sequencing and spatial transcriptomics can delineate cellular heterogeneity and cell–cell interactions within the injured microenvironment post-therapy, they primarily generate hypotheses (Chen et al., 2023). To rigorously test these hypotheses, the field must adopt a systematic approach to validate proposed pathways. The following checklist outlines critical experimental criteria for transitioning from pathway correlation to causation in stem cell analgesia research.

#### 5.2.1 Mechanistic validation checklist for pathway inference in stem cell analgesia research

To strengthen mechanistic rigor and avoid overinterpretation of correlative pathway changes, future studies should adopt a structured validation framework when implicating signaling pathways such as HIF-1α or ERK in stem cell-mediated analgesia and regeneration: (i) Activity-based confirmation rather than protein abundance alone. Changes in protein expression or phosphorylation are insufficient to establish pathway activation. For example, HIF-1α involvement should be supported by transcriptional activity assays (e.g., HIF-responsive reporter constructs or upregulation of canonical target genes such as VEGFA), rather than relying solely on protein stabilization. (ii) Loss-of-function perturbation to establish necessity. Pharmacological inhibition (e.g., pathway-specific inhibitors) or genetic approaches (siRNA/shRNA knockdown, CRISPR-based deletion) should be employed to determine whether blocking the pathway attenuates the therapeutic effect. Demonstrating that analgesic or regenerative benefits are diminished when HIF-1α or ERK signaling is inhibited provides stronger causal evidence than association alone. (iii) Temporal resolution to distinguish priming from downstream signaling. Time-resolved experimental designs are essential to clarify whether pathway activation occurs upstream (e.g., during stem cell preconditioning) or downstream (e.g., in recipient target cells). For instance, ERK inhibition could be applied during stem cell preconditioning to assess effects on secretome composition (e.g., VEGFA induction), and separately in recipient endothelial cells to test whether ERK activation is required for proliferation or tube formation. (iv) Contextual dependency and cell-type specificity. Because signaling pathways may function differently in stem cells versus target tissues (e.g., microglia, neurons, endothelial cells), pathway perturbation experiments should be performed in both compartments where feasible to establish hierarchical relationships. Adopting such criteria would help transition the field from pathway correlation toward causal mechanistic inference, thereby enhancing translational reliability.

Although many reports nominate candidate paracrine mediators (e.g., VEGF/FGF-family cytokines and chemokines) using targeted assays, candidate-based profiling is inherently limited and may overlook unexpected key effectors. The field would benefit from unbiased secretome characterization, including proteomics and multiplex cytokine/chemokine panels, complemented by quantitative validation (e.g., ELISA/Luminex) for prioritized mediators. Importantly, discovery-driven profiling should be coupled with functional perturbation (neutralization or receptor blockade) to test necessity rather than mere association. A recurring limitation in mechanistic *in vitro* studies is the reliance on single, arbitrarily chosen concentrations without formal dose–response characterization, which hampers biological interpretation and cross-study reproducibility. Future work should routinely include multi-dose designs to establish dose dependency and define effective ranges. In addition, advanced *in vitro* platforms such as three-dimensional (3D) culture systems and organoid-based models may help bridge persistent mechanistic gaps between simplified cell assays and *in vivo* complexity. Compared with conventional two-dimensional cultures, 3D systems better recapitulate tissue architecture, extracellular matrix interactions, oxygen gradients, and cell–cell communication, thereby providing a more physiologically relevant context to interrogate signaling pathways such as HIF-1α and ERK. Such platforms may facilitate more accurate assessment of pathway activity, secretome composition, and functional dependency prior to *in vivo* validation (Chacón et al., 2025).

### 5.3 Personalized stem cell therapies and combination treatment strategies

AI can analyze multi-omics data to predict patient response and optimize therapy (Aziz et al., 2025). Combination with biomaterials and targeted drug delivery [concepts akin to advanced vaccine design (Priyanka et al., 2023b)] can enhance precision. This biomaterials-enabled concept is particularly well illustrated in wound healing, where hydrogel-based delivery platforms can enhance the local retention, stability, and sustained release of stem cell–derived EVs/exosomes, thereby amplifying pro-angiogenic and pro-repair effects in chronic wounds (e.g., diabetic ulcers). Recent studies and reviews have highlighted hydrogel–exosome systems and other advanced scaffolding strategies as promising translational directions for refractory wound repair and vascularized tissue regeneration (Wang Z. et al., 2025; Liu et al., 2024; Deng et al., 2025).

### 5.4 Standardized evaluation systems and multicenter collaborative research

To overcome the barriers imposed by methodological heterogeneity (as outlined in Table 4), establishing unified preclinical evaluation standards and promoting multicenter collaborative research are essential pathways for advancing stem cell analgesia and regeneration research toward clinical application. Adopting Common Data Elements (CDEs), standardized behavioral batteries inclusive of affective pain measures, and rigorous study design (blinding, power analysis) are essential. Multicenter trials using shared protocols are needed for validation (Yerofeyeva et al., 2023). The principles of rigorous animal model use in surgery and implant research are directly relevant here (Choudhary, 2025). In line with the need for improved methodological rigor, a standardized core behavioral battery should be considered for stem cell analgesia studies. Such a battery may include at minimum: (i) one evoked hypersensitivity measure (e.g., von Frey), (ii) one spontaneous pain indicator (e.g., facial grimace or burrowing), and (iii) one affective component assay where feasible (e.g., CPP/CPA). Establishing common behavioral standards would facilitate cross-study comparability and improve translational interpretability.

**Table 4:** Standardization gaps and proposed framework for preclinical evaluation.

Evaluation domain	Current gaps and inconsistencies	Proposed elements for a standardized framework	Goal of standardization	References
Pain behavior assessment	Variations in testing methods (e.g., different mechanical stimulators), protocols, and scoring criteria across labs. Susceptibility to operator and environmental bias.	Adopt unified, automated behavioral testing platforms (e.g., dynamic plantar aesthesiometer). Establish consensus on testing timepoints post-injury/intervention.	Improve objectivity, reproducibility, and cross-study comparability of pain relief data.	Teixeira-Santos et al. (2023), McClements et al. (2020), Tappe-Theodor et al. (2019)
Molecular & histological markers	Inconsistent selection of inflammatory cytokines, neuropeptides, and regenerative markers (e.g., SOX9, Col2a1). Variability in immunohistochemical protocols and interpretation.	Define a core panel of minimally required molecular markers for specific disease models (e.g., IDD, SCI). Standardize antibody validation, staining protocols, and quantitative image analysis methods.	Ensure consistent biological endpoint measurement and mechanistic insight across different research groups.	Zhang et al. (2014), Lee et al. (2023), Tappe-Theodor et al. (2019)
Imaging analysis	Lack of standardized quantitative parameters for modalities like MRI T2 mapping in cartilage/disc studies. Inconsistent timepoints for longitudinal monitoring.	Establish guidelines for imaging acquisition parameters and quantitative analysis metrics (e.g., region-of-interest definition, signal intensity thresholds).	Enable reliable, non-invasive longitudinal assessment of tissue structure and repair in a comparable manner.	Ronis et al. (2020), Bryden et al. (2015), Kan et al. (2022)
Data reporting & collaboration	Heterogeneity in reporting cell preparation details, delivery parameters, and experimental outcomes limits data integration.	Promote use of CDEs and reporting checklists (e.g., based on ARRIVE guidelines). Foster multicenter studies with shared protocols.	Facilitate meta-analysis, data sharing, and validation of findings, accelerating the path to clinical translation.	Cao et al. (2021), Won et al. (2021)

This table summarizes the prevailing inconsistencies in preclinical evaluation and proposes core elements for a standardized framework, aiming to enhance reproducibility and accelerate the translation of stem cell therapies for pain and regeneration.

Keywords: Stem cell analgesia; Tissue regeneration; Preclinical models; Spinal cord injury; Neural stem cells; Extracellular vesicles.

## 6 Conclusion

Preclinical research on stem cell analgesia and regeneration has elucidated promising mechanisms, primarily involving paracrine-mediated immunomodulation, trophic support, and microenvironment remodeling. However, the field is constrained by significant model heterogeneity, methodological inconsistencies, and a frequent over-reliance on correlative evidence to support causal mechanistic claims. Critical appraisal of study quality, including model validity, sample size, and potential biases, is often lacking. While EVs emerge as a potentially safer alternative, their translation requires solving distinct manufacturing and standardization challenges. The path toward meaningful clinical translation in the short term likely focuses on well-defined patient populations (e.g., focal neuropathic pain, early degenerative joint/disc disease) using localized delivery routes (e.g., intrathecal, intra-articular, intradiscal). To advance, the field must prioritize: (1) developing and employing more comprehensive animal models that better capture spontaneous and affective pain; (2) conducting rigorous dose–response and mechanistic studies to distinguish robust findings from hypotheses; (3) establishing standardized evaluation frameworks to improve reproducibility; and (4) fostering multidisciplinary collaboration integrating stem cell biology, pain research, bioengineering, and AI-driven analytics. By addressing these challenges, stem cell-based therapies can move closer to realizing their potential for providing effective, long-lasting relief from chronic pain and facilitating functional tissue repair.
